# Determinants of glycemic control in female diabetic patients: a study from Iran

**DOI:** 10.1186/1476-511X-9-83

**Published:** 2010-08-11

**Authors:** Zeinab Ghazanfari, Shamsaddin Niknami, Fazlollah Ghofranipour, Bagher Larijani, Hamid Agha-Alinejad, Ali Montazeri

**Affiliations:** 1 Department of Health Education, Tarbiat Modares University, Tehran, Iran; 2 Endocrine and Metabolism Research Center, Shariati Hospital, Tehran University of Medical Sciences, Tehran, Iran; 3 Department of Exercise Physiology, Islamic Azad University, Central Tehran Branch, Tehran, Iran; 4 Department of Mental Health, Institute for Health Sciences Research, ACECR, Tehran, Iran

## Abstract

**Background:**

Since microvascular and macrovascular complications are reduced through strict glycemic control, this study carried out to identify the factors that affect glycemic control.

**Methods:**

A cross-sectional design was carried out to examine the role of demographic, anthropometric, clinical and other relevant characteristics in a sample of 103 female diabetic patients in Tehran, Iran. Personal interviews were conducted to collect data. Then blood sampling collected and the patients were divided into two outcome groups (controlled and uncontrolled diabetes). The groups were compared on the basis of their characteristics using both univariate and multivariate analyses.

**Results:**

In all 103 patients were entered into the study. The mean age of patients was 46.38 (SD = 11.42) years. Overall, the mean value of HbA1c for the whole sample was 7.5 (SD = 2.35) and 56.3% had HbA1c ≥ 7%. The findings obtained from univariate analysis revealed that there were no significant differences between controlled and uncontrolled patients. However, in multivariate analysis the waist circumference was found to be a significant predictor of increased level of HbA1c (OR = 1.04, 95% CI = 1-1.08, P = 0.04).

**Conclusions:**

The findings suggest that increased level of HbA1c is associated with waist circumference that is a modifiable factor. It seems that physical activity might be a solution to overcome this health problem. A larger study to identify other factors also is recommended.

## Background

Glycemic control is essential in diabetes management [1]. Since lower level of blood glucose leads to decreased rates of morbidity and mortality, maintaining glycemic control is a goal for all patients with diabetes [2]. Prospective randomized clinical trials and epidemiological studies have shown that glycemic control is related with reduced rates of retinopathy, nephropathy, neuropathy and cardiovascular diseases [1-3]. Glycemic control is considered as the main therapeutic goal for prevention of organ damage and other complications of diabetes [4].

Glycosylated hemoglobin (HbA_1C_) is the primary target of glycemic control. In this regard, desirable value for HbA_1C _is values below 7 [2,3]. HbA1c is a gold standard in analysis of patients' status, and is essential to ensure the optimal care of diabetic patients [5]. HbA_1C _is the index that indicates the average blood glucose during the past 3 months. One percent change in HbgA_1C _is equivalent to an approximately 35 mg/dl change in mean plasma glucose [2,6]. Smaller values of HbA_1C _indicate better glycemic control [7]. The research has shown that with each one percent reduction in the value of HbA1C, the risk of microvascular complications is reduced by 40 percent [2].

Despite evidences that strict glycemic control could reduce microvascular and macrovascular complications [8-10], a high proportion of patients remain poorly controlled [11]. Achieving optimal glycemic control in clinical practice is difficult and the reasons for its poor control are complex. A variety of factors are identified in influencing glycemic control including age, sex, education, marital status, BMI, smoking, diabetes duration, and type of medications [4,12-14]. However, the results are not consistent and in most instances more than half of the variance in HbA1c changes are not explained [13]. Thus, the current study aimed to examine these factors in Iran, where there are no information on the topic. We thought this might contribute to existing knowledge and help to enhance women's health.

## Methods

### Design and data collection

A cross-sectional study was carried out to examine the role of demographic, anthropometric, clinical and other relevant characteristics in glycemic control among women attending a diabetes clinic affiliated to the Charity Foundation for Special Diseases (CFFSD), in Tehran, Iran between November 2008 and July 2009. The inclusion criteria were: aged between 15 to 70 years, being literate, no history of diabetes complications, and mental and disabling disorders. To collect data, trained interviewers carried out face-to-face interviews.

### Measures

1. Demographic information: this included data on age, education, marital status, smoking, duration of disease, and family history of diabetes mellitus

2. Anthropometrics data: this included information on body mass index (BMI), and waist and hip circumference. Trained research personnel measured height and weight by a Seca 220 (made by Germany) while the subjects were minimally clothed and not wearing Shoes. The body mass index was calculated based on heights and weights [BMI = weight (kg)/(height (m))^2^]. Based on the BMI, women were grouped into different categories as recommended by the WHO: normal range (BMI = 18.5-24.9 kg/m^2^), overweight (BMI = 25-29.9 kg/m^2^), and obese (BMI ≥ 30 kg/m^2^) [15].

Waist and hip circumference were measured in centimeters using a plastic tape meter at the level of the umbilicus and of the greater trochanters.

3. Clinical measures: this included data on glycosylated hemoglobin (HbA_1C_), systolic and diastolic blood pressure, the presence of dislipidemia, and medication. Glycosylated hemoglobin was analyzed using immunoturbidometric assay [16]. Blood pressure was measured with a mercury sphygmomanometer in a sitting position after 5 minutes rest.

4. Other relevant data: this included information on self-monitoring blood glucose (SMBG).

### Statistical analysis

Patients were divided into two groups for comparison: (1) controlled: the HbA1c level less than 7%, (2) uncontrolled: the HbA1c level equal or greater than 7% [2,3]. Both univariate and multiple logistic regression analyses were used to indicate the association between dependent (controlled vs. uncontrolled diabetes) and independent variables. Independent variables tested for an association were age, education, marital status, smoking, body mass index, waist and hip circumferences, duration of disease, family history of diabetes mellitus, SMBG, medication type, systolic and diastolic blood pressure, and the presence of dislipidemia.

### Ethics

The Ethics Committee of Tarbiat Modares University approved the study. Written informed consent was obtained from participants after comprehensive explanation of the procedure involved.

## Results

In all, 103 patients were entered into the study (45 controlled and 58 uncontrolled diabetes). The mean age of participants was 46.38 (SD = 11.42), and ranged from 15 to 68 years. The mean for BMI and duration of disease were 27.7 (SD = 5.04), and 8.55 (SD = 6.10) respectively. Overall, the mean value of HbA1c for the whole sample was 7.5 (SD = 2.35) and 56.3% had HbA1c ≥ 7%. The characteristics of the study sample are presented in Table 1.

**Table 1:** The results obtained from univariate logistic regression analysis for increased level of HbA1c

	Controlled diabetes (n = 45, HbA1c < 7)	Uncontrolled diabetes (n = 58, HbA1c ≥ 7)		
	**No (%)**	**No (%)**	**OR (95% CI)**	**P-value**

**Age **(year; Mean, SD)	45.3 (10.98)	46.84 (12.48)	1.01 (0.98-1.04)	0.51

**Education**				

Higher	9 (20)	7 (12.1)	1.0 (ref.)	

Secondary	29 (64.4)	40 (69)	1.77 (0.59-5.31)	0.31

Primary	7 (15.6)	11 (19)	2.02 (0.51-7.94)	0.31

**Marital status**				

Married	38 (84.4)	44 (75.9)	1.0 (ref.)	

Single	4 (8.9)	7 (12.1)	1.51 (0.41-5.56)	0.53

Widowed/divorced	3 (6.7)	7 (12.1)	2.01 (0.49-8.34)	0.33

**Smoking**				

No	41 (91.1)	52 (89.7)	1.0 (ref.)	

Yes	4 (8.9)	6 (10.3)	1.18 (0.31-4.47)	0.80

**Body Mass Index **(kg/m^2^)				

< 25	19 (43.2)	16 (27.6)	1.0 (ref.)	

25-30	15 (34.1)	22 (37.9)	1.74 (0.68-4.43)	0.24

> 30	10 (22.7)	20 (34.5)	2.37 (0.87-6.52)	0.09

**Family history of diabetes**				

Yes	36 (80)	44 (75.9)	1.0 (ref.)	

No	9 (20)	14 (24.1)	1.27 (0.49-3.28)	0.62

**SMBG**				

Yes	32 (71.1)	37 (63.8)	1.0 (ref.)	

No	13 (28.9)	21 (36.2)	1.40 (0.60-3.23)	0.43

**Medication type**				

None	4 (8.9)	1 (1.7)	1.0 (ref.)	

Insulin alone	10 (22.2)	6 (10.3)	2.40 (0.21-26.82)	0.47

Tablet and insulin	5 (11.1)	7 (12.1)	5.60 (0.47-66.45)	0.17

Tablet alone	26 (57.8)	44 (75.9)	6.77 (0.72-63.86)	0.09

**Dislipidemia**				

No	22 (50)	31 (53.4)	1.0 (ref.)	

Yes	22 (50)	27 (46.6)	0.87 (0.40-1.91)	0.73

**Duration of disease** (year; Mean, SD)	8.66 (6.49)	8.76 (5.74)	1.003 (0.94-1.07)	0.93

**Systolic Blood pressure **(mmHg; Mean, SD)	119.67 (17.63)	122.07 (16.33)	1.01 (0.98-1.03)	0.47

**Diastolic Blood pressure **(mmHg; Mean, SD)	76.11 (11.12)	77.33 (8.39)	1.01 (0.97-1.06)	0.52

**Waist circumference** (Cm; Mean, SD)	85.52 (11.24)	89.44 (10.24)	1.03 (0.1-1.07)	0.07

**Hip circumference** (Cm; Mean, SD)	101.65 (10.71)	103.92 (9.19)	1.02 (0.98-1.07)	0.25

CI: Confidence Interval

The results obtained from univariate logistic regression analysis indicated that there were no significant differences between controlled and uncontrolled patients. The results are shown in Table 1. However, when forward conditional multiple logistic regression analysis was performed, the results showed that waist circumference emerged as a significant factor for increased level of HbA1c (OR = 1.04, 95% CI = 1-1.08, P = 0.04).

## Discussion

This was the first study to examine the association between glycemic control and demographic, anthropometric, clinical and other relevant data in a sample of Iranian females with diabetes. Overall the proportion of patients with poor glycemic control was high (56.3%), and controlled and uncontrolled patients did not differ significantly with respect to age, education, marital status, smoking, duration of disease, family history of diabetes mellitus, SMBG, medication, systolic and diastolic blood pressure, waist and hip circumferences and the presence of dislipidemia. However, the association between increased level of HbA1c and variables studied were in the expected directions (Table 1).

The results obtained from multivariate logistic regression analysis indicated that the differences between patients who gained control and those who did not were due to waist circumference (OR = 1.04). Similarly Yoshida and Okosun argued that physiologic factors are important in diabetes control where they have showed the association between glycemic control and waist circumference [17,18]. However, Hartz et al. suggested that patient factors such as understanding of diabetes and adherence to recommended behaviors and not physiologic factors are primary important factors on gaining control over glycosylated hemoglobin [12].

Any modifiable factors that influence glycemic control could be important [19]. In present study waist circumference was a predictor of increased level of HbA1c and thus it is the only modifiable factor in this regard. Waist circumference is often used as a proxy measure of abdominal adipose tissue, in particular, visceral adipose tissue (VAT) in clinical settings. VAT has been reported to create a greater risk for developing obesity related disorders than subcutaneous adipose tissue (SAT) [20-23]. Obesity should be considered as a chronic disease with multi-factorial etiology, and treatment must be maintained for life-first with lifestyle interventions (energy-reduced diet and increased physical activity) and then with pharmacologic approaches, when necessary [24].

Although other studies found a significant relationship between age [25-27], BMI [26], smoking [26,28], duration of diabetes [4,27,29], and HbA1c, this study did not show such associations. However, our findings on relationship between duration of diabetes, and medication and HbA1c level was similar to the findings by Goudswaard et al. and Hartz et al., respectively [13,12].

The findings from this study might be influenced by several limitations. The sample size was small making it difficult to generalize the findings. Secondly, patients were female and recruited from a single institute rather than being a community-based sample. Thus the findings could not be generalized beyond this study sample. Finally most measures in this study were self-reported and therefore the possibility of recall bias should not be neglected.

## Conclusions

The findings suggest that increased level of HbA1c is associated with waist circumference that is a modifiable factor. It seems that physical activity might be a solution to overcome this health problem. A larger study to identify other factors also is recommended.

## Competing interests

The authors declare that they have no competing interests.

## Authors' contributions

ZGh was the main investigator, collected the data, performed the statistical analysis, and drafted the manuscript. SHN supervised the research and contributed to all aspects of the study. FGH was advisor of the study and contributed to design and study implementation. BL was advisor of the study and helped to recruit the patients. HAA helped in implementation of the study and interpretation of the data. AM helped as a consultant in study design, questionnaire, and revised the final article. All authors read and approved the final manuscript.
